# Alternative models for academic family practices

**DOI:** 10.1186/1472-6963-6-38

**Published:** 2006-03-20

**Authors:** J Lloyd Michener, Truls Østbye, Victoria S Kaprielian, Katrina M Krause, Kimberly SH Yarnall, Susan D Yaggy, Margaret Gradison

**Affiliations:** 1 Department of Community and Family Medicine, Duke University Medical Center, Durham, USA

## Abstract

**Background:**

The Future of Family Medicine Report calls for a fundamental redesign of the American family physician workplace. At the same time, academic family practices are under economic pressure. Most family medicine departments do not have self-supporting practices, but seek support from specialty colleagues or hospital practice plans. Alternative models for academic family practices that are economically viable and consistent with the principles of family medicine are needed. This article presents several "experiments" to address these challenges.

**Methods:**

The basis of comparison is a traditional academic family medicine center. Apart of the faculty practice plan, our center consistently operated at a deficit despite high productivity. A number of different practice types and alternative models of service delivery were therefore developed and tested. They ranged from a multi-specialty office arrangement, to a community clinic operated as part of a federally-qualified health center, to a team of providers based in and providing care for residents of an elderly public housing project. Financial comparisons using consistent accounting across models are provided.

**Results:**

Academic family practices can, at least in some settings, operate without subsidy while providing continuity of care to a broad segment of the community. The prerequisites are that the clinicians must see patients efficiently, and be able to bill appropriately for their payer mix.

**Conclusion:**

Experimenting within academic practice structure and organization is worthwhile, and can result in economically viable alternatives to traditional models.

## Background

The Future of Family Medicine Report [1] calls for a fundamental redesign of the American family physician workplace. This change will require additional time, effort, and financial investment [2], at a time when academic family practices are under heavy economic pressure. Departments of family medicine are caught between an increasingly uninsured patient population, high costs of resident education, and practice plan cost structures based on high-margin subspecialty practices [3,4].

Most departments have given up hope of operating self-sustaining practices, and seek support from their specialty colleagues in return for referrals and revenue generated by ancillary services. Such relationships are possible, as each dollar of revenue generated in the family practice can be linked to $8 of revenue within a health system [5]. Still, the process of seeking and defending this support is politically tiresome, and may compromise the ideals of family medicine. It also leaves little latitude for experimentation with new models of practice.

Alternative models of operating academic family practices that do not require practice plan subsidies would be highly beneficial, especially if they were consistent with the goals and principles of family medicine. This article presents a series of alternative clinical office programs created by one family medicine department to address these challenges. The programs use a consistent accounting system, which allows for comparison across programs.

## Methods

The basis for comparison for the experiments is the traditional academic family medicine practice at Duke University, staffed primarily by family physician faculty, family medicine residents, physician assistants and nurse practitioners. It had been operating under the auspices of the faculty practice plan, common to all departments in the School of Medicine, which provides highly efficient billing and collection services. However, as with most academic family practices [3], the center had consistently operated at a deficit, and required ongoing subsidies from the plan, the hospital, and/or the department.

The continuing deficits caused considerable attention to be focused on operating efficiencies, including patient flow, staffing ratios, and payer mix. Practice efficiencies eventually exceeded Medical Group Management Association (MGMA) private practice norms for work units per clinician full-time equivalent, and below median staffing. However, over a period of more than 10 years, the practice was never able to entirely cover its cost of operation.

A financial statement from the best performing year in that decade is shown in Table 1 (column 1), concluding with a $270,000 (9%) deficit. Resident salaries were paid by the hospital, and are excluded, but the costs of precepting and education are included, as are the collections from the residents' patient care. It was clear that, as structured, the model practice could not be operated without subsidy.

## Results

Since 1998, the department has undertaken a number of "experiments" with different practice types and alternative models of service delivery. The intent of these was to develop practices that met the needs of the surrounding community, and could be financially sustainable.

### Suburban multispecialty faculty practice

The first experiment was modest – to join in the academic health center's expansion efforts and establish a suburban family practice in a multi-specialty office (Table 1, column 2). Located in a fast-growing area near a large shopping center, this practice was designed to appeal to the relatively young, commercially insured population living and working nearby. Despite a better payer mix, this practice was financially unsuccessful. High space costs ($55/sq.ft.) combined with lower than expected visits led to substantial losses. This family practice office was closed within 3 years.

### Small community clinic

The next experiment sought to provide care to an underserved neighborhood near the university, with high proportions of individuals uninsured or insured through Medicaid (Table 1, column 3). Discussions were opened with a nearby federally qualified community health center (CHC) about operation by the department as a satellite clinic of the CHC, staffed by two physician assistants and a nurse, and supported by a member of the family medicine faculty and a staff assistant. A contract between the CHC and the department allowed the department to hire the personnel and pay for the time of the faculty physician, with the clinical operations billed through the CHC at a per-visit rate, regardless of payer source. The clinic is able to charge patients on the CHC's sliding scale fee, making health care affordable for indigent patients. Space for the clinic was secured within a city-managed neighborhood center, where access for the neighborhood was easy, and overhead costs were low.

The new office, now in operation for close to two years, incorporates many of the aspects of the new model of family practice [1]. The office provides a personal medical home for its target population, 65% of whom are new Latino immigrants. Patients are 74% uninsured, and 19% are covered by Medicaid. The practice provides patient-centered care, eliminates barriers to access, and provides care in the community context. Furthermore, it uses an electronic health record developed at Duke, and focuses on quality and safety. Learners rotate through the clinic on an elective basis, but no learners are based at the site. As shown in Table 1, high volume and low overhead, combined with per-visit CHC reimbursement for both Medicaid and uninsured visits, and electronic records linked to the main information system of the academic health center, allow the small clinic to provide care in a previously underserved – or even *un*served – community [6].

The community clinic has already shown clear evidence of value to the health system: a 2004 survey of clinic patients, with a 96% response rate, revealed that of the patients who came to the clinic as drop-ins (52% of visits), 40% said they would have gone to the emergency room if the clinic were not available. Emergency department diversion for uninsured patients represents a significant cost savings for the health system. As the clinic continues to grow, and establishes its patient panel, improved (though still deficit) operations are forecast for its third year.

### School-based clinics

A third initiative was the establishment of clinics in 3 elementary public schools and a public high school in Durham. These clinics reach children and adolescents that are rarely seen in the traditional office. Each clinic is operated in donated space within the school with no overhead costs assessed to the department. The high school clinic is staffed by a nurse practitioner (NP) and a full-time nurse; the elementary school clinics are each staffed by a half-time NP and a receptionist whose time is donated by the school district. All clinics are supervised by off-site faculty physicians and all provide limited, elective learner participation. A financial summary of the high school clinic (Table 1, column 4) shows a small operating deficit, primarily related to lower billable visit rates and the absence of the per-visit fee from the CHC to cover indigent students.

The school clinics serve as an alternative source of care than the emergency department. Responding to a 2004 survey, parents of students indicated that they would have taken the child to the emergency department for 8% of the visits, which amounts to 127 emergency department visits averted. In addition, 89% of the elementary school clinic visits and 95% of the high school clinic visits resulted in the child's return to class, rather than being sent home. As a result, the school clinics have been continued, with support from the health system.

### Home-based senior care

A three-year-old senior health program places a geriatric nurse practitioner and a physician assistant with geriatric experience in senior public housing sites, with oversight from a senior faculty family physician and support from 2 staff assistants. The program staff includes 3 social workers, contracted through the county social services agency, who provide in-home case management and help enroll patients in Medicaid and other public programs (financial summary from the initial project site is in Table 1, column 5). Residents rotate through the site as part of the community health rotation; it is also the site for care by the community health fellow. Billing is again through the federally qualified community health center, this time with a per-visit rate for Medicaid patients, and a nominal rate for uninsured patients. This is an 'office-free' model of care, now serving 362 low-income, medically fragile seniors in their own homes. The practice has a high level of patient acceptance and can break even at 9 visits per day. After three years of operation, the program is nearing this point.

By lowering the barrier to seeking care, acute conditions may be treated earlier and chronic conditions can be monitored closely, with hospital admissions and nursing home stays avoided. The program has recently expanded to 10 senior housing projects around the county. State Medicaid data for 2003 and 2004 show that, for patients enrolled in this senior program during the most recent two-year period, ambulance costs decreased 49%, emergency room costs decreased 41%, and in-patient hospital costs decreased 68%, while prescription costs, costs to the Community Alternative Plan for the Disabled (CAP-DA), and home health costs increased [7].

### Reforming the traditional practice

Finally, the core teaching family practice was moved out of the faculty practice plan in 2002 and is now operating as a hospital-based facility (Table 1, column 6). The costs of operating the office are now incurred by the hospital, which bills a facility fee for each visit. Professional charges are billed by the practice plan but at a reduced rate, which varies based on the contracts held by the hospital. This shift to a hospital-based facility has had only a minor effect on patient volume and throughput, and produced minimal changes in payer mix. It has, however, constituted a substantial economic shift, with the family practice no longer requiring financial subsidy.

Based on the earlier experiments that used physician assistants and nurse practitioners as providers, team-based care has also been instituted. Faculty physicians are now co-leaders with midlevel providers on teams that include other physicians and midlevel providers, residents, nurses, and nursing assistants. Reception staff, a social worker and a pharmacist support the teams. Expansion of services within the practice is now underway, including the addition of a dietitian, physical therapist, and health educator.

## Discussion

The models reported here have a common financial reporting system, and common faculty. The hospital-based practice demonstrates that a financially self-sufficient academic practice is possible, while the others show that care delivery to disadvantaged populations can be conducted at costs similar to those of a teaching practice. Nonetheless, the results may not be easily generalizable. Setting up and operating these new offices and clinics required a high degree of cross-institutional cooperation and trust. Crafting contracts that included academic departments and the practice plan, as well as hospitals, public schools, county social services, the city housing authority and a federally qualified community health center, required a level of institutional oversight and assistance which may be difficult to find in other settings. Yet, by challenging conventional wisdom, the models may encourage others to try their own experiments.

Having an appropriate avenue for billing is particularly important. In the models described here, operations and billing occurred under the aegis of a federally qualified community health center, an academic hospital, or an academic practice plan, as appropriate. Given the wide range of payers in the local community, and the range of reimbursement for identical visits, it was essential to identify and work with the operational unit that most closely matched the needs of the population served. For the family medicine center, the change from a faculty practice plan to a hospital-based clinic with both professional and facility fees allowed an already efficient practice to break even or better. The successful transition, however, was predicated on the willingness of the major payers to reimburse the facility fee charges.

Space cost was another variable, often ignored, that became a critical success factor. Costs for space varied from $0 to $55 per net square foot, which led to a difference in cost per visit of $22 to $101. For a practice close to breaking even, the cost of space can easily determine financial sustainability. Lower cost office space, or no space at all, can make a large difference.

Practice efficiency may be one of the greatest challenges. Overall, academic family practices see fewer patients, and generate fewer relative value units per provider, than their peers in private practice [8]. In part, this may reflect an inherent inefficiency involved in the education of students and residents. However, the resident problem can be exaggerated. In the settings described here, faculty work units per full-time-equivalent (4217) were on par with the national mean for private practice (4244, MGMA) [8]. Paradoxically, part of the higher productivity stems from the educational setting: residents are scheduled in sets of four per half day, mixed across the classes, so that each preceptor is responsible for more patients, and more work units, than would be possible if she were personally seeing patients. In addition, the family practice has a goal of being like a private practice in operation – of being "a practice that teaches" rather than a "teaching practice." With about 70% of visits with faculty and less than 30% with residents, and resident time clustered to maximize precepting efficiency, achieving private practice efficiencies is possible. Similarly, residents have been included in the community-based sites, but only after the practices were operating efficiently and community acceptance was achieved.

Finally, the centrality of midlevel practitioners has become clear. Highly trained physician assistants (PAs) and nurse practitioners (NPs), with physician support for the more complicated patients, provide excellent care at a cost per visit lower than that of a physician working alone. While it is not clear what the optimal mix is of physicians to midlevel providers, our experience has been that the financial performance of the team increases with the number of PAs and NPs, but that the need for interaction and supervision will restrict a single physician from working with more than 2–4 midlevel providers. It has not yet been possible to test the model beyond a configuration of two midlevel providers per one physician, however, due to a shortage of qualified PAs and NPs.

## Conclusion

Family medicine departments in the United States face many challenges. Even when run efficiently, academic family practices can be financial burdens. But not all have to lose money – academic family practices can, at least in some settings, operate without subsidy. The prerequisites are that the practices must see patients efficiently, and be able to bill appropriately for their payer mix. Family practices can also help patients by offering accessible and affordable community-based alternatives. By creatively weaving together community resources to provide much-needed services to our underserved patients, family medicine departments can incorporate many of the elements called for in the Future of Family Medicine Report, and can demonstrate to skeptical learners and faculty that innovation in care delivery is possible.

## Competing interests

The author(s) declare that they have no competing interests.

## Authors' contributions

JM conceived of the study, and its design and drafted parts of the manuscript. TØ and VK participated in the design of the study, drafted parts of the manuscript and edited the mansucript. KK, KY, SY and MG helped in drafting and editing of the manuscript. JM, KK and SY oversaw data collection and management. All authors read and approved the final manuscript.

## Pre-publication history

The pre-publication history for this paper can be accessed here:



## Figures and Tables

**Table 1:** Department of Community and Family Medicine clinic models

	**Family Practice** ** Clinic (FPC)**	**Multi-specialty** ** Clinic**	**Small Community** ** Clinic**	**High School** ** Clinic**	**Home-based** ** Senior Care**	**FPC** ** (Hospital-based)**
						
Personnel						
Physician FTE	5.60	1.50	0.10	0.05	0.20	5.60
Midlevel FTE	1.25	-	2.15	1.00	2.20	1.25
FM Resident FTE	4.30	.20	-	-	-	4.30
Administrative FTE	1.00	-	-	-	-	1.00
Nursing FTE	17.00	-	1.00	1.00	-	17.00
Support FTE	23.00	-	1.00	-	2.00	23.00
						
Characteristics						
Visits/Procedures	46,966	6,790	7,644	3,843	4,320	46,344
Visits/FTE	4,201	3,994	3,397	3,660	1,792	4,145
Billing	FPP	FPP	FQHC	FPP	FQHC	FPP+H
						
Revenue*						
Professional Fees	2,954	596	268	75	322	3,014
Technical Fees	-	-	-	-	-	1,290
Total	2,954	596	268	75	322	4,304
						
Expenses*						
Provider Personnel	1,417	262	267	91	222	1,700
Other Personnel	1,277	145	34	40	67	1,277
Non-personnel	529	303	29	7	57	1,017
Other Reimbursements	-	-	-	-	-	(973)
Total	3,224	710	330	138	346	3,021
						
Net to Department*	(269)	(115)	(63)	(63)	(24)	297
						
Net to Hospital*	-	-	-	-	-	986

FPP -Faculty Practice Plan

FPP+H -Faculty Practice Plan and Hospital

FQHC -Federally Qualified Health Center.

* numbers in thousands.

The hospital reimbursement and net to the hospital for the Hospital based Family Practice are estimates.

A separate accounting for those items specific to this clinic is not available.
